# Cell Cycle Arrest and Cytotoxic Effects of SAHA and RG7388 Mediated through p21^WAF1/CIP1^ and p27^KIP1^ in Cancer Cells

**DOI:** 10.3390/medicina55020030

**Published:** 2019-01-29

**Authors:** Umamaheswari Natarajan, Thiagarajan Venkatesan, Vijayaraghavan Radhakrishnan, Shila Samuel, Periannan Rasappan, Appu Rathinavelu

**Affiliations:** 1 Rumbaugh-Goodwin Institute for Cancer Research, Nova Southeastern University, Fort Lauderdale, FL 33314, USA; ubaruthrarini@gmail.com or un15@mynsu.nova.edu (U.N.); tvenkatesan@nova.edu (T.V.); 2 VRR Institute of Biomedical Science, Kattupakkam, Chennai 600056, India; drrvijayaraghavan@gmail.com (V.R.); vrribms@gmail.com (S.S.); vrrdiagnostic@gmail.com (P.R.); 3 College of Pharmacy, Health Professions Division, Nova Southeastern University, Fort Lauderdale, FL 33314, USA

**Keywords:** SAHA, RG7388, MDM2, p53, p21, cell cycle arrest, cell death

## Abstract

*Background and Objective*: Alterations in gene expressions are often due to epigenetic modifications that can have a significant influence on cancer development, growth, and progression. Lately, histone deacetylase inhibitors (HDACi) such as suberoylanilide hydroxamic acid (SAHA, or vorinostat, MK0683) have been emerging as a new class of drugs with promising therapeutic benefits in controlling cancer growth and metastasis. The small molecule RG7388 (idasanutlin, R05503781) is a newly developed inhibitor that is specific for an oncogene-derived protein called MDM2, which is also in clinical trials for the treatment of various types of cancers. These two drugs have shown the ability to induce p21 expression through distinct mechanisms in MCF-7 and LNCaP cells, which are reported to have wild-type TP53. Our understanding of the molecular mechanism whereby SAHA and RG7388 can induce cell cycle arrest and trigger cell death is still evolving. In this study, we performed experiments to measure the cell cycle arrest effects of SAHA and RG7388 using MCF-7 and LNCaP cells. *Materials and Methods*: The cytotoxicity, cell cycle arrest, and apoptosis/necroptosis effects of the SAHA and RG7388 treatments were assessed using the Trypan Blue dye exclusion (TBDE) method, 3-(4,5-dimethylthiazol-2-yl)-2,5-diphenyltetrazolium bromide (MTT) assay, fluorescence assay with DEVD-*amc* substrate, and immunoblotting methods. *Results:* The RG7388 treatment was able to induce cell death by elevating p21^WAF1/CIP1^ through inhibition of MDM2 in LNCaP, but not in MCF-7 cells, even though there was evidence of p53 elevation. Hence, we suspect that there is some level of uncoupling of p53-mediated transcriptional induction of p21^WAF1/CIP1^ in MCF-7 cells. *Conclusion*: Our results from MCF-7 and LNCaP cells confirmed that SAHA and RG7388 treatments were able to induce cell death via a combination of cell cycle arrest and cytotoxic mechanisms. We speculate that our findings could lead to the development of newer treatments for breast and prostate cancers with drug combinations including HDACi.

## 1. Introduction

Breast cancer is one of the most common causes of cancer-related death worldwide. Despite continued efforts around the globe, there have been only marginal improvements in breast cancer treatment success. The median survival time for patients with metastatic breast cancer is no more than one year [1]. The five-year survival rate for prostate cancer patients with distant metastasis is only around 30%, versus the 99% survival rate for patients with localized disease [2]. Therefore, the need for new drugs and efficient strategies for the effective treatment of aggressive cancers continues to exist. Although the development of cancer is triggered by a complex process of tumorigenesis, cancers are intimately linked to gene mutations and abnormal gene expressions. Therefore, epigenetic changes, which are known to affect gene transcription and translation without modifying the underlying DNA sequence, are also very common and significant in the process of tumorigenesis. In normal cells, the histones are modified via acetylation, methylation, and phosphorylation to meet certain needs of the cellular function [3,4]. Acetylation of histone is one of the most common epigenetic modifications that is regulated largely by histone acetyltransferases (HATs), which transfer the hydrophobic acetyl group from acetyl coenzyme A to specific lysine residues on the N-terminal tails of histones H2A, H2B, H3, and H4 [5].

The addition of acetyl groups to histones leads to the neutralization of positive charges of the amino groups and increases the steric interference that can loosen the histone-DNA structure and make it more accessible for transcription machinery. On the other hand, the DNA stretching and activation of the gene expression can be turned off when histone is deacetylated [6,7]. Histone deacetylation is carried out by enzymes that belong to two families, the classical histone deacetylase (HDAC) family and the SIR2 family [8]. In total, there are 11 HDAC isoenzymes that deacetylate histones within the nucleus, and the specific HDACs are differentially regulated to modulate the expression of various groups of genes [9]. When a histone deacetylase inhibitor such as suberoylanilide hydroxamic acid (SAHA) is used, the gene expression can be activated. Enhanced expression of tumor suppressors and checkpoint inhibitors such as p21^WAF1/CIP1^ and p27^KIPl^ can lead to suppression of cell differentiation, growth arrest, apoptosis, and autophagy, indicating that the net effect of the histone acetylation blockade is inhibition of cell proliferation [10,11,12]. Hence, several HDACi, including SAHA (also known as vorinostat), have stimulated enthusiasm in the field of oncology, with more than 40 clinical trials initiated to date. In fact, when HDAC inhibitors are combined with other inhibitors, including blockers of DNA methyl transferases, it was reported that the combination increased antitumor effects on myelodysplastic syndrome and prostate, ovarian, and pancreatic endocrine tumor cells, suggesting superior benefits for carefully selected combinations including HDAC inhibitors [13].

Another gene that is important in cell cycle regulation, tumor growth, and angiogenesis is MDM2 [14]. In several metastatic sarcomas, the MDM2 gene has been shown to encode a protein in the molecular weight range of 90 KD (p90), which can form an oligomeric complex with both mutant and wild-type p53 proteins. The interaction between MDM2 and p53 is believed to occur through direct binding by involving a specific binding domain on the MDM2 protein [15,16,17]. In addition, it has been well established that MDM2 contains ubiquitin ligase (E3) activity in its carboxy terminus, which is responsible for the polyubiquitination of p53 and consequent proteasomal degradation [18]. Initially it was believed that only the full-length MDM2 protein (p90) could act as a negative regulator in cells that contain genetically intact p53 genes and abolish cell cycle control mechanisms. However, studies on MDM2 splice variants, including ones lacking p53 binding domains, have suggested that mechanisms other than p53 suppression could also be involved in promoting malignancy during MDM2 gene amplification [19,20]. So far, more than 40 alternatively spliced variants of MDM2 gene transcripts have been identified in various tumor cells, but it is unknown at this time whether all of the splice variants are translated into protein. In fact, some of them have been detected in only a few particular tumor types, suggesting that they might even contribute to the phenotype transformation of tumor cells, whereas others, such as MDM2-A and MDM2-B, may be associated with tumorigenesis in general [21]. So far, our preliminary studies have clearly suggested that MDM2 protein may be critically involved in the transcription control of vascular endothelial growth factor (VEGF) expression in several cancers and promote tumor angiogenesis [22,23].

Among the various strategies, blocking the direct interaction of MDM2 with p53 protein is considered the most beneficial. Traditionally, disrupting protein-protein interaction was considered a difficult task, due to the large binding interface of the protein partners. However, the small interface of MDM2-p53 made it possible to design small-molecule inhibitors such as RG7388 to target the MDM2- p53 interaction. Although very little progress was made in the first few years after deriving the crystal structure of the MDM2-p53 complex, lately, several classes of small-molecule inhibitors with distinct chemical structures and properties have been reported. An actual breakthrough in the design of small-molecule inhibitors for MDM2 was achieved through the discovery of Nutlin by Vassilev and colleagues [24]. However, many of the initially designed molecules were not utilized in clinical trials because of limited in vivo potency, poor bioavailability, and excessive toxicity [25]. Keeping the initial setback in mind, a new generation of MDM2 inhibitors was developed to optimize potency and bioavailability. In this regard, the RG7388 small molecule belongs to the new generation of MDM2 inhibitors with good selectivity and improved bioavailability [26]. Several studies that were conducted by utilizing RG7388 to effectively rescue p53 and activate downstream apoptotic pathways in p53 wild-type cell lines have yielded interesting results [27]. However, the detailed mechanisms of cell cycle arrest and cell death induced by the HDAC inhibitor SAHA and the MDM2 inhibitor RG7388 have not been fully established. Moreover, the HDACi function of SAHA is not limited to modifying the acetylation status of histones, but is also suspected to be involved in the modification of non-histone proteins including p53. Therefore, a possible interaction or synergy between HDACi and MDM2 inhibitors was speculated. The current study provides some interesting insights into the mechanisms mediated by these two inhibitors, which strongly impact p21 expression.

## 2. Materials and Methods

### 2.1. Reagents

The histone deacetylase (HDAC) inhibitor SAHA was purchased from Selleckchem (Houston, TX, USA) and the MDM2 inhibitor RG7388 was purchased from MedChemExpress (Monmouth Junction, NJ, USA). The primary antibodies against p53, p21, p27, aurora kinase-B (AURK-B), cell division cycle 25C (CDC25C), cyclin dependent kinase 1 (CDK1), Bax, Bak, and cleaved poly (ADP-ribose) polymerase (PARP) (1:1000) were purchased from Cell Signaling Technology, (Danvers, MA, USA). MDM2 antibody (1:500 mouse monoclonal) was purchased from Santa Cruz Biotechnology, (Dallas, TX, USA). β-actin (1:2000) was purchased from Sigma Aldrich (St. Louis, MO, USA). The secondary antibodies anti-rabbit, anti-mouse, and horseradish-peroxidase (HRP) conjugated and dimethylsulfoxide (DMSO) were purchased from Sigma Aldrich. Nitrocellulose membrane (0.45 µm) was purchased from Amersham (GE Healthcare Life Sciences, Marlborough, MA, USA). ECL was purchased from KPL Biosolutions (Milford, MA, USA). DEVD-*amc* CellEvent^TM^ Caspase-3/7 Green ReadyProbe^TM^ was purchased from Thermo Fisher (Molecular Probes, Life Technologies, Carlsbad, CA, USA). Other chemicals and reagents used in this experiment were of research grade.

### 2.2. Cell Culture and Drug Treatment

The MCF-7 cells (human breast adenocarcinoma cell line) was obtained from American Type Culture Collection (Manassas, VA, USA). The LNCaP cells (human prostate adenocarcinoma) used in our experiments were a generous gift from Dr. Thomas Powell (Cleveland Clinic Foundation, Cleveland, OH, USA). The MCF-7 and LNCaP cells were cultured with Dulbecco’s Modified Eagle’s Medium (DMEM) and RPMI-1640, supplemented with 10% fetal bovine serum (FBS), 1% L-glutamine, 1.5 g/L sodium bicarbonate, 1% amphotericin B, and 1% penicillin G-streptomycin. The cells used in our experiments were carefully maintained with 95% air and 5% CO_2_ at 37 °C in a humidified atmosphere. When MCF-7 and LNCaP cells reached 75–80% confluency, they were treated with 7.5 µM of SAHA and 2.0 µM of RG7388 for 24 h. After incubation, the cells were used for protein extraction and Western blot analysis. Similarly, cell viability assays and fluorescence staining were also performed after treating the cells with the above mentioned procedure.

### 2.3. Cell Viability Assessment Using MTT and Trypan Blue Dye Exclusion Method

The MCF-7 and LNCaP cells were plated at a density of 5 × 10^3^ cells/well in 96-well plates and incubated at 37 °C under 95% air and 5% CO_2_ for 24 h. When the cells reached 75–80% confluency, they were treated for 24 h with different concentrations of the drugs. After incubation, the viability of the cells was assessed using TBDE and MTT assay. In the TBDE method, after removing the incubation medium, equal parts of 0.4% trypan blue dye were added to the cell suspension. The analysis mixture was incubated for less than 3 min at room temperature. The viability of the cells was counted using the TC20 automated cell counter from Bio-Rad (Hercules, CA, USA). In the MTT assay, the cells were seeded into a 96-well plate at a density of 5 × 10^3^ per well (200 μL) and treated with the following: control; SAHA: 0.5, 2.5, 5.0, 7.5, and 10.0 μM; and RG7388: 1.0, 2.0, 2.5, 5.0, and 7.5 µM. After 24 h of treatment, 20 μL of MTT solution (5 mg/mL in PBS) was added to each well and the cells were incubated at 37 °C for an additional 3–4 h. At the end of the specified incubation period, 200 μL of DMSO was added to each well. To solubilize the MTT-formazan precipitate, the plate was gently rotated on an orbital shaker for a few minutes. The absorbance was read at 650 nm with a Versamax microplate reader (Molecular Devices, Sunnyvale, CA, USA).

### 2.4. Protein Preparation and Western Blot Analysis

After 24 h of treatment, the cells were lysed with radio-immunoprecipitation assay (RIPA) buffer containing a protease inhibitor cocktail and sodium orthovanadate (Santa Cruz Inc., Dallas, TX, USA), for 30 min at 4 °C. Cell lysates were centrifuged at 4 °C for 20 min at 14,000 rpm to clarify the samples from unbroken cells and organelles. The concentrations of proteins in the clarified samples were determined by using the bicinchoninic acid (BCA) protein assay method (Thermo Fisher Scientific, Grand Island, NY, USA). When the protein samples were analyzed by Western blot using 7.5–12% sodium dodecyl sulfate-polyacrylamide gel electrophoresis (SDS-PAGE), equal concentrations of proteins were loaded into the wells and were also verified later with β-actin levels. After transfer of proteins, the membranes were blocked using 5% nonfat dry milk and then probed with specific antibodies: MDM2, p53, p21, p27^Kip1^, AURK-B, CDC25C, CDK1, Bax, Bak, cleaved PARP, and β-actin. Finally, detection of specific protein bands on the membranes was achieved by incubating in a solution containing LumiGLO Reserve chemiluminescent substrate (KPL, Milford, MA, USA). Densitometric analyses were performed using the ImageJ program (National Institutes of Health, Bethesda, MD, USA).

### 2.5. Fluorescence Imaging for Cell Death Assessment

The fluorescent caspase substrate DEVD-*amc* is a cell-permeant caspase-3/7 substrate that consists of a 4-amino acid peptide (DEVD) conjugated to a nucleic acid-binding dye, *amc* (7-amino-4-methylcoumarin). The peptide sequence is based on the PARP cleavage site Asp^216^ for caspase-3/7. Uncleaved DEVD-*amc* is intrinsically nonfluorescent when it is not bound by the DNA. During apoptosis, caspase-3 and caspase-7 proteins are activated and the conjugate is cleaved so that free dye can stay intracellular and bind to DNA. Thus, cleavage of the caspase-3/7 recognition sequence labels the apoptotic cells, generating a bright green fluorescence. Once cleaved from DEVD, the *amc* that is bound to DNA can be excited at 502 nm to emit fluorescence that can be measured at 535 nm. To determine the effects of the drugs, the cells were treated with SAHA or RG7388 for 24 h. After the drug treatment, the cells were washed and incubated with the caspase-3/7 green DEVD-*amc* substrate for 15–30 min. The fluorescence in the apoptotic cells was measured using a Victor 3 spectrofluorometer.

### 2.6. Statistical Analysis

The data are presented as the mean ± standard deviation (SD) from statistical significance between the groups, as analyzed by one-way analysis of variance (ANOVA), followed by a least significant difference (LSD) test. *p* < 0.05 was considered statistically significant.

## 3. Results

### 3.1. Changes in Cell Viability by SAHA and RG7388 Treatments in MCF-7 and LNCaP Cells

**Figure 1:**
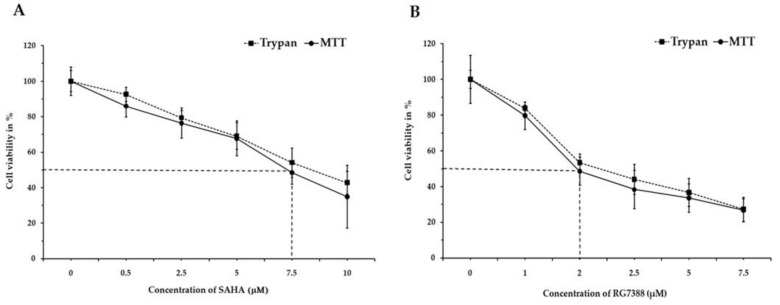
Assessment of cell viability using Trypan Blue dye and MTT assay on MCF-7 cells after treatment with (**A**) SAHA and (**B**) RG7388. The effect of 24 h treatment on MCF-7 cell viability was assessed using 0.5, 2.5, 5.0, 7.5, and 10.0 µM concentrations of SAHA and 1.0, 2.0, 2.5, 5.0, and 7.5 µM concentrations of RG7388. Data are presented as mean ± SD from a minimum of three independent experiments.

**Figure 2:**
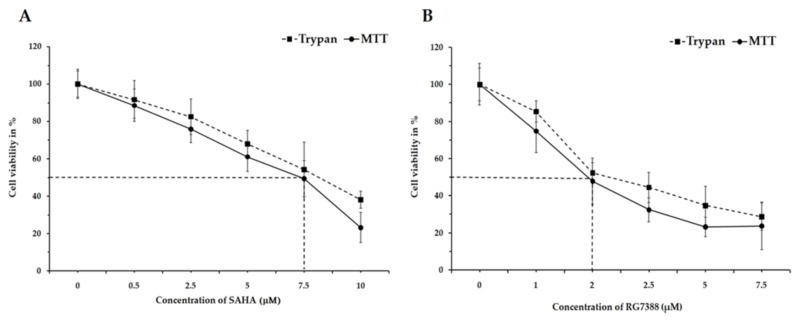
Assessment of cell viability using Trypan Blue dye and MTT assay on LNCaP cells after treatment with (**A**) SAHA and (**B**) RG7388. The effect of 24 h treatment on LNCaP cell viability was assessed using 0.5, 2.5, 5.0, 7.5, and 10.0 µM concentrations of SAHA and 1.0, 2.0, 2.5, 5.0, and 7.5 µM concentrations of RG7388. Data are presented as mean ± SD from a minimum of three independent experiments.

Inducing programmed cell death (apoptosis) through cell cycle arrest is one of the major goals of cancer treatment. In this regard, our studies were designed to specifically analyze the intracellular events following SAHA and RG7388 treatments using MCF-7 and LNCaP cells. During the cell viability assessment experiments, both the TBDE and MTT assay methods were employed. Treatment with SAHA and RG7388 produced significant reduction in cell viability after 24 h, as can be seen in Figure 1 and Figure 2. The treatment effects of SAHA are shown in Figure 1A and Figure 2A. The half maximal inhibitory concentration (IC_50_) for SAHA after 24 h of treatment was found to be 7.5 µM for MCF-7 and LNCaP cells. On the other hand, RG7388 treatment produced stronger cytotoxic effects, therefore the IC_50_ value was found to be 2.0 µM in MCF-7 cells after 24 h of treatment (Figure 1B). In LNCaP cells, RG7388 treatment produced similar IC_50_ values (Figure 2B).

### 3.2. Effect of SAHA and RG7388 Treatments on p21^WAF1/CIP1^ and p27^Kip1^ Levels

**Figure 3:**
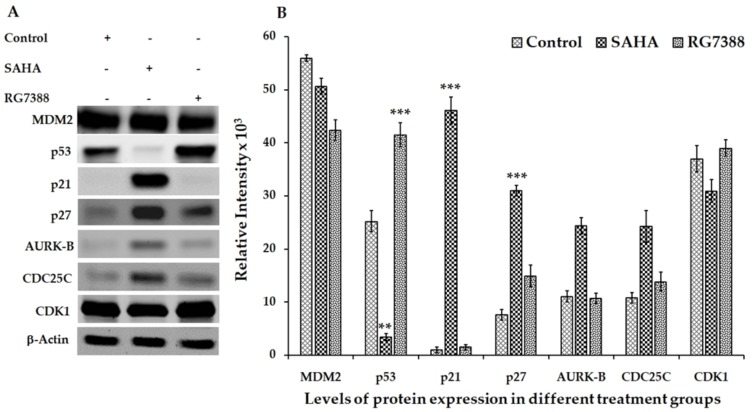
Effect of SAHA and RG7388 treatments on cell cycle-related protein levels in MCF-7 cells. (**A**) Figure shows upregulation of p21 and p27 levels after treatment with 7.5 µM concentration of SAHA. However, RG7388 treatment was able to elevate p27 levels significantly while the p21 level remain unchanged. In fact, a slight downregulation of MDM2 was seen in MCF-7 cells, while the p53 level was elevated after treatment with RG7388. (**B**) The right panel shows the results of relative band intensity of Western blot bands measured using ImageJ software. The normalization of protein levels is depicted using the band intensities of β-actin bands. Data are presented as mean ± SD from a minimum of three independent experiments. ** *p* < 0.01 vs. control; *** *p* < 0.001 vs. control.

**Figure 4:**
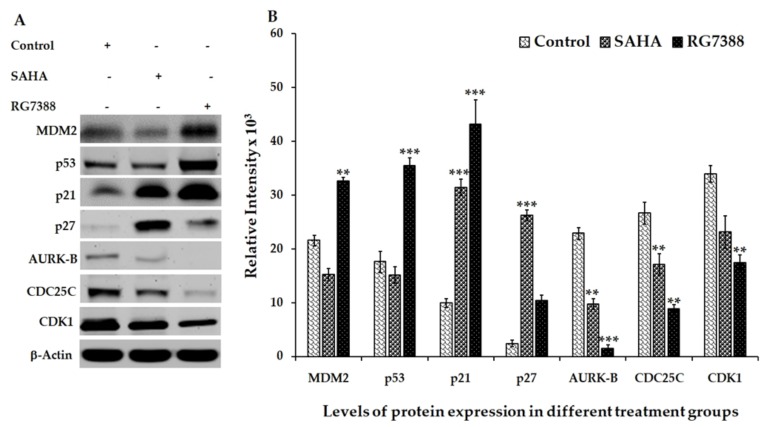
Effect of SAHA and RG7388 treatments on cell cycle-related protein levels in LNCaP cells. (**A**) Upregulation of p53, p21, p27 and MDM2 levels was seen after treatment with 2.0 µM concentration of RG7388, and upregulation of p21 and p27 was seen after treatment with 7.5 µM concentration of SAHA. Downregulation of AURK-B, CDC25C, and CDK1 levels were seen after treatment with SAHA and RG7388. (**B**) The right panel shows the results of relative band intensity of the Western blot bands measured using ImageJ software. The normalization of protein levels is depicted using the band intensities of β-actin bands. Data are presented as mean ± SD from a minimum of three independent experiments. ** *p* < 0.01 vs. control; *** *p* < 0.001 vs. control.

Based on the results shown in Figure 3 and Figure 4, SAHA and RG7388 induced strong cell cycle arrest in both MCF-7 and LNCaP cells. Treatment of MCF-7 cells with SAHA showed significant elevation of p27^Kip1^ (Figure 3). On the other hand, treatment of LNCaP cells with SAHA and RG7388 showed elevated levels of both p21^WAF1/CIP1^ and p27^Kip1^ (Figure 4). In LNCaP cells, the elevation of p21^WAF1/CI^ caused by SAHA was only around 1.3-fold, whereas RG7388 treatment induced a 3.5-fold increase. However, treatment of LNCaP cells with SAHA did not elevate either MDM2 or p53 protein levels even after 24 h of treatment. It is interesting that in LNCaP cells, SAHA was able to induce p21^WAF1/CIP1^ expression without any significant change in the expression level of p53. As expected, RG7388 treatment elevated both MDM2 and p53 levels by nearly 95% and 120%, respectively, in the LNCaP cells (Figure 4A,B). The results observed with MCF-7 cells were quite interesting, because SAHA treatment was able to increase p21^WAF1/CIP1^ levels even though the p53 level was significantly lowered in MCF-7 cells. On the other hand, the RG7388 treatment did not elevate p21 levels in MCF-7 cells. However, RG7388 was able to significantly elevate both p53 levels and p21 in LNCaP cells (Figure 3A,B). Expectedly, SAHA treatment increased p27 and p21 levels while lowering the AURK-B, CDC25C and CDK-1 levels compared to the untreated LNCaP cells. In addition to the p21^WAF1/CIP1^ levels, the results of the p27^Kip1^ levels following SAHA treatment suggested an interesting mechanism in both cell lines. The elevation of p27^Kip1^ was nearly 300% in both LNCaP and MCF-7 cells after 24 h of treatment. Though RG7388 treatment elevated p27^Kip1^ levels in both cell lines, the increase was lower than what was seen with SAHA treatment, and it was only around 90% in those cell lines. Thus, our results clearly suggest that SAHA treatment could significantly elevate p21^WAF1/CIP1^ and p27^Kip1^ expression in both cell lines, while RG7388 treatment was able to markedly elevate p21^WAF1/CIP1^ but only slightly elevate p27^Kip1^ expression in LNCaP cells. Such elevation following RG7388 treatment was completely absent in MCF-7 cells.

### 3.3. Aurora Kinase-B (AURK-B), CDC25C, and CDK1 Levels in SAHA and RG7388 Treated Cells

In addition to the expression results of cyclin-dependent kinase inhibitors (CDKIs) discussed in the previous section, significant decreases of AURK-B, CDC25C, and CDK1 were also noted in LNCaP cells following both SAHA and RG7388 treatment (Figure 4A,B). However, the changes in the levels of these cell cycle regulators were marginal with RG7388 treatment in MCF-7 cells, while SAHA treatment showed noticeable elevation of AURK-B and CDC25C levels (Figure 3A,B). This observation suggests that the mechanisms of cell cycle arrest and cell death in these two cell lines are distinct, with sufficient cross-talk between the cell cycle and apoptosis pathways involved.

### 3.4. Apoptotic Effects of SAHA and RG7388 Treatments on MCF-7 and LNCaP Cells

**Figure 5:**
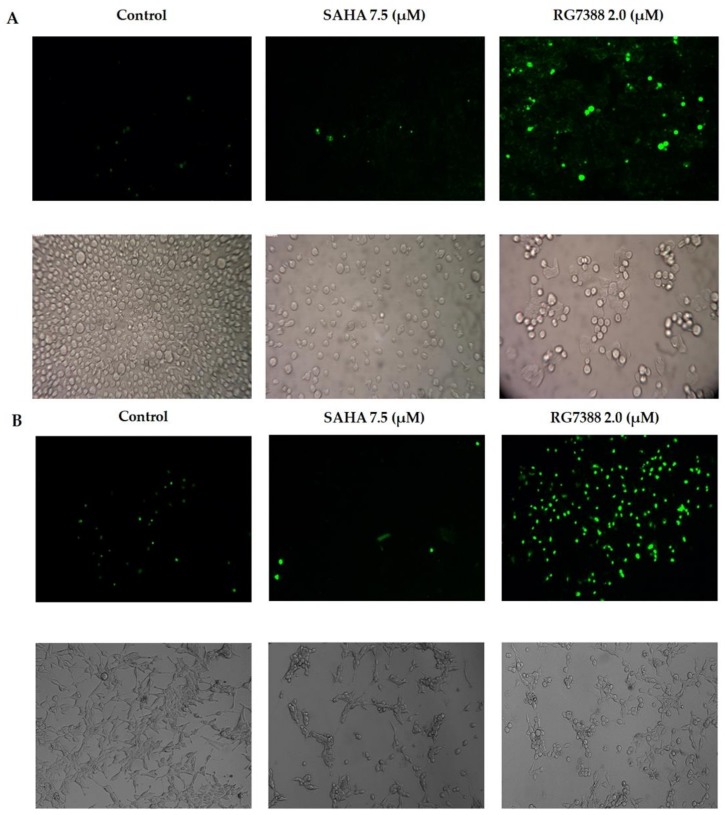
Apoptotic cell death in (**A**) MCF-7 and (**B**) LNCaP cells, shown by fluorescence images obtained using DEVD-*amc*. The results of cell death induced by SAHA and RG7388 treatment for 24 h are shown in the upper panels. The effects of treatment on cell death observed by light microscopy imaging are shown in the lower panels.

**Figure 6:**
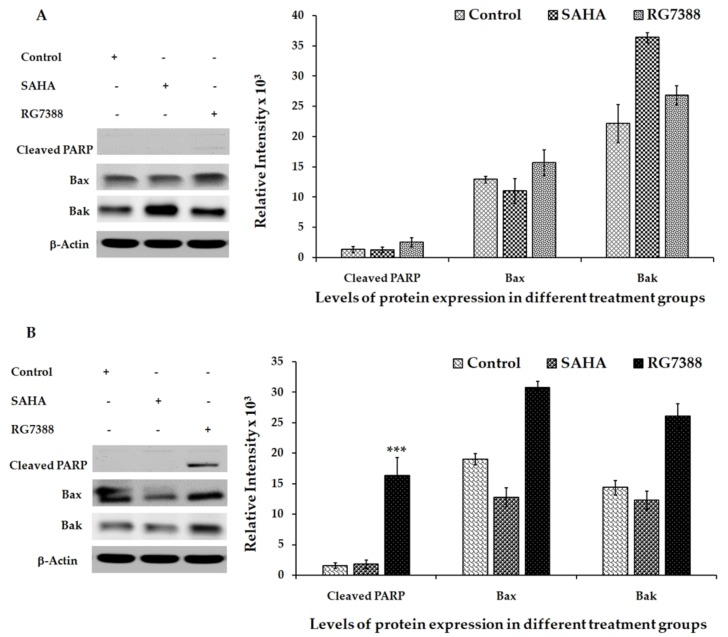
Apoptotic markers detected by Western blot analysis of Bak, Bax, and cleaved PARP expressions in treated (**A**) MCF-7 and (**B**) LNCaP cells. Density of the Western blot bands in the right panel was quantified using ImageJ software. Data are presented as mean ± SD from a minimum of three independent experiments. Asterisks indicate a statistically significant difference. *** *p* < 0.001 vs. control.

Since the cell cycle-related proteins offered interesting findings, we determined the apoptotic effects of SAHA and RG7388 treatments using the fluorescence staining method with DEVD-*amc* fluorogenic substrates specific for caspase-3/7 (Figure 5A,B). The fluorescence substrate DEVD was employed mainly to verify the activation of the caspases, particularly caspase-3 and caspase-7, following the drug treatments. As discussed above, the cells that were treated with RG7388 produced high levels of fluorescence in both MCF-7 and LNCaP because of the cleavage of DEVD-*amc* substrate. Interestingly, SAHA treatment produced lower levels of fluorescence, possibly due to less cleavage of the substrate as a result of less activation of the apoptosis pathway. However, the light microscopy imaging of the unstained cells also showed significant reduction in cell numbers after RG7388 treatment. The results of the imaging experiments were supported by the Western blotting results of the PARP cleavage, Bax and Bak elevations, which were performed after treating the cells with RG7388 (Figure 6A,B). Interestingly, the SAHA treatment was not able to elevate Bax levels but it elevated Bak levels in MCF-7 cells. In LNCaP cells SAHA appears to produce a slight decrease in Bax and Bak levels after 24 h of treatment. In addition, SAHA treatment did not produce PARP cleavage, even after 24 h of treatment with 7.5 µM concentration (Figure 6A,B). However, SAHA treatment showed measurable levels of cell death, but lower than the extent of cell death induced by RG7388 treatment after 24 h in both MCF-7 and LNCaP cells. So far, the greater effect of RG7388 is evidenced by the elevation of PARP cleavage that coincides well with increases in the levels of Bax and Bak, suggesting activation of the intrinsic pathway by this MDM2 inhibitor leading to apoptotic cell death in the cancer cells.

## 4. Discussion

Many studies have shown that DNA damage can lead to the upregulation and activation of the p53 tumor suppressor protein, which can produce cell cycle arrest at the G-to-S transition checkpoint or lead to the induction of apoptosis through transcriptional activation of p21^WAF1/CIP1^. The p27^KIPl^ protein that is encoded by the CDKN1B gene is also known to play a significant role in inducing apoptosis. The p27^KIPl^ belongs to the CIP/KIP family of CDKI proteins and is intimately involved in regulating the progression of cells through the different phases of the cell cycle [28]. Both p21^WAF1/CIP1^ and p27^KIPl^ proteins can bind and prevent the activation of cyclin D-, E-, A-, and B-dependent kinases, in particular, and block the activation of CDK2 by cyclin E or CDK4 by cyclin D, and thus controls the cell cycle progression from the G1 to the S phase [29,30]. Hence, both p21^WAF1/CIP1^ and p27^KIPl^ are commonly referred to as cell cycle dependent kinase inhibitor (CDKI) proteins because of their ability to stop cell division using CDK inhibitory mechanisms [29]. Many of the cell cycle arrest and anticancer effects of SAHA are known to be mediated through transcriptional induction of the p21^WAF1/CIP1^ gene and elevation of its protein levels. It has been demonstrated that SAHA-induced p21^WAF1/CIP1^ promoter activity is primarily through two Sp1 sites located at positions 782 and 769, relative to the transcription start site, in a p53-independent manner [31]. While Sp1 and Sp3 are the major transcription factors binding to the Sp1 site of the p21^WAF1/CIP1^ promoter and induce gene expression, it was reported that SAHA did not alter their DNA binding activities. Since the induction of p21^WAF1/CIP1^ during SAHA treatment is mostly independent of p53, the acetylation-dependent mechanism is generally considered to be responsible for the observed induction of p21^WAF1/CIP1^ expression in cells that lack functional p53. In addition, SAHA has been reported to induce hyperacetylation of the histones, which was shown to transcriptionally upregulate the expression of p27^KIPl^ to cause co-inhibitory effects [32]. As result of the cell cycle arrest and apoptosis mediated death of cancer cells, regression of tumors in in vivo models has been reported [33]. In further confirmation of the mechanistic ability of the CDKIs, the levels of p27^KIPl^ were found to be high in the quiescent cells and low in the cancer cells, due to sequestration into the cyclin D-CDK complexes. Therefore, the removal of p27^KIPl^-mediated repression of CDK2 or CDK4 was reported to allow for the progression of cells through the G-S checkpoint more rapidly. The importance of p27^KIPl^ in regulating the cell cycle was further confirmed by the fact that mice that were null for CDKN1B grew faster, exhibited organomegaly, and had a higher incidence of pituitary tumors in comparison to age-matched controls [34,35,36]. It has been previously reported that overexpression of p27^KIPl^ in cancer cells could result in strong G-to-S arrest and therefore be more cytotoxic to cancer cells relative to the effects of overexpression of p21^WAF1/CIP1^ [37]. Similarly, through the study results presented here, we demonstrate the mechanism of p21^WAF1/CIP1^- and p27^KIPl^- induced cell cycle arrest and its possible role in inducing apoptosis during SAHA and RG7388 treatment.

**Figure 7:**
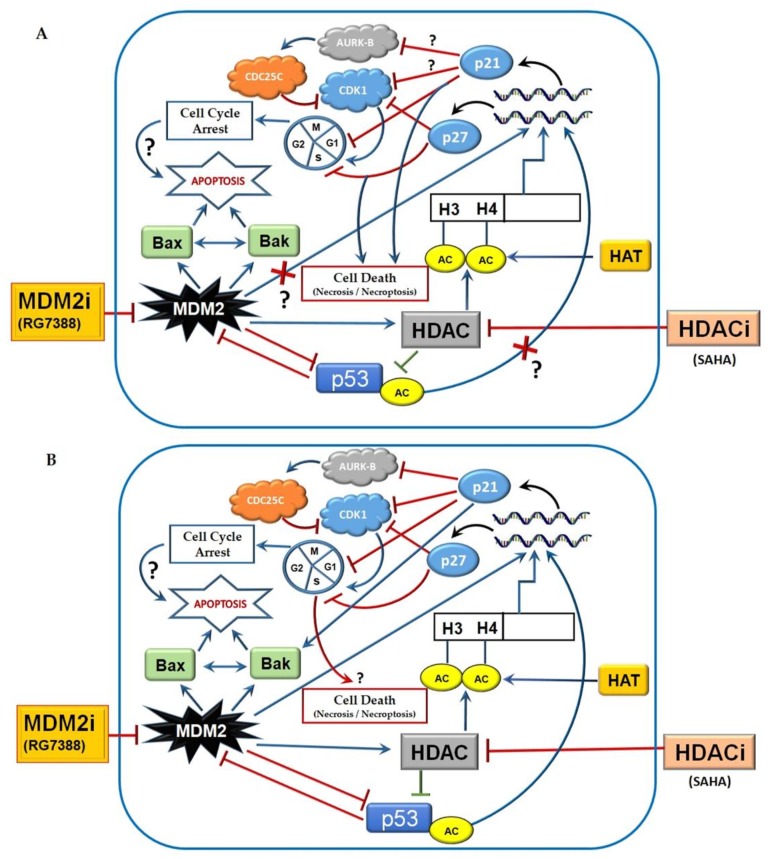
Pathways impacted by SAHA in (**A**) MCF-7 and (**B**) LNCaP cells, illustrating the pathways activated by SAHA in MCF-7 cells leading to cell death, and the cycle arrest and apoptosis pathway triggered by MDM2 inhibitor (RG7388) and HDACi (SAHA) in LNCaP cells, respectively.

So far, it has been well established that histone deacetylase inhibitors such as SAHA can kill transformed cells or cancer cells that are in cultures or implanted in animal models [38]. There are several HDACi that have progressed from preclinical testing to clinical use in the last few years. In this regard, US Food and Drug Administration (FDA) approval of HDAC inhibitors such as vorinostat, romidepsin, belinostat, and panobinostat for cancer treatment has promoted HDAC inhibitors as effective therapeutics for the treatment of various cancers. Another strategy of using HDACi as chemosensitizers to increase the efficiency of other chemotherapeutic compounds is also showing great potential for better therapeutic outcomes in preclinical and clinical trials. In addition, the use of a combination of drugs that target DNA repair pathways and HDACi holds great promise [39]. In a recent study, when LNCaP cells were treated with increasing concentrations of HDAC-3-specific inhibitor RGFP966, the androgen-mediated proliferation of LNCaP cells was significantly inhibited [40]. Interestingly, in the same study, they were able to show inhibition of the proliferation of the androgen-independent 22Rv1 cell line, suggesting that HDAC-3-specific inhibitor treatment could be very useful for treating castration-resistant prostate cancers (CRPCs), in which the constitutively active androgen receptor (AR) splice variant AR-V7 is expressed and dominant over wtAR [41]. Moreover, this study further demonstrated that SAHA, not RGFP966, induced the expression of snail family zinc finger 2 (SLUG), zincfinger E-box-binding homeobox 1 (ZEB1), and vimentin, which are the key epithelial-to-mesenchymal transition (EMT) markers in both 22Rv1 and LNCaP cells [40]. Thus, HDAC inhibitors exhibit a variety of cell cycle arrest and pro-apoptotic mechanisms that are effective even in CRPC cell lines. Similarly, RG7388 also produced cell cycle arrest and cell death through inhibition of the MDM2-p53 interaction and p21^WAF1/CIP1^ elevation in our experiments. It is fully evident that RG7388 effects are mediated through p53, because the X-ray crystal structures of the N-terminal of MDM2 that complexes with the N-terminal of p53 reveal that the binding site of p53 in MDM2 is formed by 14 residues: Leu^54^, Leu^57^, Ile^61^, Met^62^, Tyr^67^, Gln^72^, Val^75^, Phe^86^, Phe^91^, Val^93^, His^96^, Ile^99^, Tyr^100^, and Ile^101^. MDM2 has a deep hydrophobic cleft on which the p53 protein, after adopting an α-helical conformation, interacts primarily through three hydrophobic residues, Phe^19^, Trp^23^, and Leu^26^ of p53, which are buried deep in the cleft. To effectively disrupt the interaction of MDM2 with p53, the inhibitor molecules must mimic the three hydrophobic interactions that are involved in their binding. Molecular modelling and simulation studies have so far revealed that 4-chlorophenyl and the neopentyl group of RG7388 occupy the Trp^23^ and Phe^19^ pockets, while its 3-chlorophenyl group occupies the Leu^26^ pocket and participates in an additional π-π interaction with the His^96^ residue. In addition, the pyrrolidine Cα carbonyl was shown to form a hydrogen bond with the NH of His^96^ [27]. Although RG7388 and some of the other MDM2 inhibitors, such as Nutlin-3, have a shared mechanism of action, RG7388 has superior potency and specificity for MDM2 compared to the other MDM2 inhibitors. The greater potency of RG7388 is probably responsible for the stronger cell cycle arrest and cell death effects observed. However, in MCF-7 cells, there was only p27^KIPl^ elevation, and there was no noticeable elevation of p21^WAF1/CIP1^ or Bax to induce apoptosis. This interesting observation suggests that the RG7388-induced cell death in MCF-7 cells is probably executed by p27^KIPl^ through mechanisms that do not require Bax elevation (Figure 7A). However, in LNCaP prostate cancer cells, SAHA and RG7388 were able to significantly induce cell cycle arrest through elevation of p21^WAF1/CIP1^ and p27^KIPl^ that led to apoptosis (Figure 7B). In this process of executing apoptotic cell death, elevated Bax and Bak leading to PARP cleavage appears to be intimately involved following treatment of LNCaP cells with RG7388. Another interesting observation from our experiments was the differential abilities of SAHA and RG7388 to induce cell cycle arrest and cell death. Very interestingly, SAHA was able to elevate p21^WAF1/CIP1^ expression without elevating p53 levels in either MCF-7 or LNCaP cells. As expected, inhibition of MDM2 with RG7388 induced p21^WAF1/CIP1^ expression by reactivating p53 in LNCaP cells due to strong inhibition of MDM2 [42]. Similarly, MDM2 inhibition with MI-219 was shown to elevate p21 levels and produce p53-dependent sensitization of LNCaP prostate cancer cells to radiation, antiandrogen therapy, and the combination of the two. These findings support MDM2 small-molecule inhibitor therapy as an intensification strategy to improve clinical outcomes in high-risk localized prostate cancers [43]. Accordingly, a clinical trial was conducted in the United Kingdom with idasanutlin (RO5503781), another inhibitor of MDM2, alongside abiraterone and prednisolone or enzalutamide, and that combination was expected to help men with prostate cancer who were no longer responding to hormone therapy [44].

Several earlier studies analyzed the status and role of cyclins D and E in growth-arrested prostate cancer cells and pointed to the distinct classes of genes that might be involved in the regulatory process of prostate cancer cell growth [45,46,47,48,49,50,51,52]. Another important master switch in the cell cycle regulation is Rb, and its status of phosphorylation parallels cell transit through G1 into the S phase [53,54]. An important mechanism that regulates Rb phosphorylation and controls the cell cycle progression is through cyclin D and the CDK4/6 complex. The activated cyclin D/CDK4 complex can phosphorylate Rb and prevent its inhibitory binding to E2F, facilitating the entry of cells from G1 into S phase [55]. It appears that RG7388 treatment might inhibit CDK1 by blocking the AURK-B pathway that also involves CDC25C. However, the majority of the cell cycle arrest effects of p21^WAF1/CIP1^ are believed to be mediated through inhibition of Rb phosphorylation. Hence, the role of Rb cannot be ruled out in mediating cell death caused by SAHA treatment. However, results of another study demonstrated very clearly that drugs such as celecoxib could increase the expression of p27^KIPl^ proteins and induce cell death. Interestingly, the study with celecoxib showed that the rate of apoptosis was higher with the activation of p27^KIPl^. Based on that evidence, it was suggested that upregulation of p27^KIPl^ may contribute to the induction of cell cycle arrest and apoptosis even when p21^WAF1/CIP1^ levels are not altered [28,56]. In addition to their intracellular function as CDKIs, both p21^WAF1/CIP1^ and p27^KIP1^ may serve as predictive biomarkers for prostate cancer [57]. One recent study showed that low-level expression of p27 in prostate cancer biopsy samples exhibited good correlation as an independent predictor of both clinical recurrence and disease-specific survival [58]. The predictive role of p27 was confirmed in a USA-based cohort, where p27 expression in needle biopsy samples from in 161 men showed good correlation as an independent predictor of biochemical recurrence subsequent to radical prostatectomy [57]. One of the minor limitations in our study was the broad-spectrum effects of SAHA, which is known as a pan-HDAC inhibitor that targets several isoforms of the enzymes in this family. Isoform-specific HDACi should provide greater insight into the cell-specific mechanisms that were observed in our study.

## 5. Conclusions

Our results suggest that cell death caused by SAHA treatment in both cell lines was primarily through induction of p21^WAF1/CIP1^ and p27^Kip1^ levels independent of p53. In support of this conclusion, it has been reported in the literature that elevated levels of acetylated histones (H2 and H3) and consequent activation of intracellular signals, including p21^WAF1/CIP1^ elevation, are responsible for the cell cycle arrest and cell death that are typically observed in cancer cells when HDAC inhibitors are used. On the other hand, our experiments with RG7388 treatment were able to induce cell death by elevating p21^WAF1/CIP1^ through a p53-dependent mechanism in LNCaP cells through inhibition of MDM2. Interestingly, RG7388 treatment was not able to elevate p21^WAF1/CIP1^ in MCF-7 cells, even though there was evidence of p53 reactivation, as can be seen in the Western blot results. Hence, we suspect that there is some level of uncoupling of p53-mediated transcriptional induction of p21^WAF1/CIP1^ in MCF-7 cells. Thus, different drugs with varying ability to induce p21 expression exhibited distinct mechanisms of cell death through p53-dependent and -independent mechanisms. As this points to an interesting intracellular interplay between two or more cell cycle-related pathways, confirmation of the actual cell death mechanism induced by RG7388 in MCF-7 in cancer cells requires additional exploration.
